# Lactate Monitoring in Intensive Care: A Comprehensive Review of Its Utility and Interpretation

**DOI:** 10.7759/cureus.66356

**Published:** 2024-08-07

**Authors:** Pallavi Deulkar, Amol Singam, V N K Srinivas Mudiganti, Abhishek Jain

**Affiliations:** 1 Critical Care Medicine, Jawaharlal Nehru Medical College, Datta Meghe Institute Of Higher Education and Research, Wardha, IND

**Keywords:** clinical outcomes, biomarker, shock, sepsis, intensive care, lactate

## Abstract

Lactate monitoring is critical in managing critically ill patients in intensive care settings. Elevated lactate levels often signify underlying metabolic disturbances such as tissue hypoxia, anaerobic metabolism, or impaired lactate clearance, which are prevalent in conditions like sepsis, shock, and trauma. Understanding the physiological basis of lactate production and its significance in clinical practice is essential for interpreting its diagnostic and prognostic value. This comprehensive review aims to explore the utility of lactate monitoring across various critical care scenarios. It provides an overview of lactate's metabolic pathways, methods of measurement, and the clinical implications of interpreting lactate levels in different contexts. Additionally, the review discusses current evidence on lactate-guided therapeutic interventions and highlights challenges and limitations to their application. By synthesizing the existing literature and clinical insights, this review aims to enhance the understanding of the role of lactate monitoring in assessing disease severity, guiding treatment strategies, and predicting outcomes in critically ill patients. Ultimately, this review underscores the importance of integrating lactate monitoring into routine clinical practice to optimize patient care and improve clinical outcomes in intensive care settings.

## Introduction and background

Lactate monitoring is indispensable in critical care, offering vital insights into the metabolic status and perfusion adequacy of patients in intensive care units (ICUs) [1]. Elevated lactate levels serve as a crucial indicator of tissue hypoxia, anaerobic metabolism, or impaired lactate clearance, conditions frequently encountered in critically ill individuals such as those suffering from sepsis, shock, or trauma. By tracking lactate levels, clinicians can promptly identify and respond to metabolic derangements, thus optimizing therapeutic interventions and potentially improving patient outcomes [2]. Lactate, produced as a byproduct of anaerobic glycolysis in various tissues, including muscles and the brain, holds significant physiological relevance. In healthy individuals, lactate is efficiently metabolized and cleared by the liver and kidneys [3]. However, in critical illness, factors such as tissue hypoperfusion or cellular dysfunction can disrupt this balance, leading to elevated lactate levels in the bloodstream. Therefore, monitoring lactate provides a direct window into the metabolic state of patients, aiding clinicians in assessing the severity of illness and guiding therapeutic decisions [4].

This review aims to provide a comprehensive exploration of lactate monitoring in intensive care settings. By synthesizing current literature and clinical practices, this review aims to elucidate the multifaceted roles of lactate as a biomarker. Specifically, it will investigate its utility in assessing disease severity, guiding therapeutic strategies, and predicting clinical outcomes in critically ill patients. Such insights are crucial for enhancing clinical decision-making processes within ICUs, potentially improving patient care and prognosis.

## Review

Physiological basis of lactate production

Metabolic Pathways Leading to Lactate Formation

**Figure 1:**
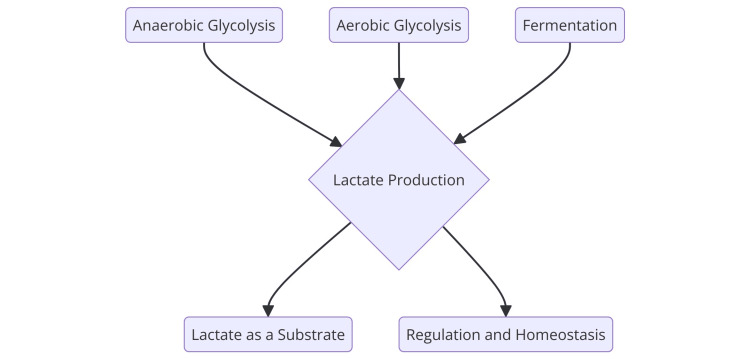
Physiological basis of lactate production Image credits: Dr Pallavi Deulkar

Lactate formation occurs through multiple metabolic pathways, predominantly involving glycolysis and the subsequent conversion of pyruvate to lactate. The most well-known pathway is anaerobic glycolysis, an oxygen-independent process. In this pathway, glucose is converted into pyruvate through glycolysis, and pyruvate is then converted to lactate by the enzyme lactate dehydrogenase (LDH). This reversible reaction favors lactate synthesis, resulting in a typical lactate-to-pyruvate ratio of 25:1 [3]. Aerobic glycolysis is another pathway leading to lactate formation. Similar to anaerobic glycolysis, aerobic glycolysis converts glucose into pyruvate. However, in this pathway, pyruvate is transported into the mitochondria, where it undergoes metabolism via the Krebs cycle and oxidative phosphorylation to produce more adenosine triphosphate (ATP). If the cytosolic rate of pyruvate formation exceeds its mitochondrial utilization, pyruvate is converted to lactate by LDH, contributing to lactate production [5]. Fermentation is another process that can produce lactate. Like anaerobic glycolysis, fermentation converts glucose into pyruvate, which is then reduced to lactate using nicotinamide adenine dinucleotide (NADH) generated during glycolysis. This oxygen-independent process enables cells to generate ATP without oxygen [6]. The Warburg effect in cancer cells exemplifies another form of aerobic glycolysis leading to lactate production. Cancer cells, especially those exhibiting the Warburg effect, preferentially utilize aerobic glycolysis to produce lactate from glucose due to mitochondrial dysfunction, which hinders their ability to use oxygen for ATP production [7]. Lactate can also be metabolized through gluconeogenesis, converting lactate back to glucose in the liver, kidneys, and heart. This conversion is part of the Cori cycle, where lactate produced in one tissue is transported to another and converted back to glucose [8]. The physiological basis of lactate production is shown in Figure 1.

Factors Influencing Lactate Levels in Critically Ill Patients

Several key factors can influence lactate levels in critically ill patients. Higher Acute Physiologic Assessment and Chronic Health Evaluation (APACHE) II and Sequential Organ Failure Assessment (SOFA) scores, which indicate greater disease severity and organ dysfunction, are independent risk factors for not achieving target lactate kinetics in these patients. The stress response also plays a significant role; an elevated heart rate, a physiological stress marker, is an independent risk factor for not reaching target lactate kinetics from six to 24 hours. Reducing the heart rate with beta-blockers may improve lactate clearance. Similarly, high blood glucose levels, another manifestation of the stress response, can negatively impact lactate metabolism and the achievement of lactate kinetic targets [9]. In critical illness, inadequate oxygen delivery, oxygen uptake, or tissue perfusion can also lead to hyperlactatemia and acidemia. Lactate is a strong predictor of mortality in critically ill patients, with the magnitude and rate of change in serum lactate levels being important. Differences in therapeutic strategies between hospitals, such as blood transfusion and inotropic therapies, can also influence lactate kinetics. Additionally, liver function and renal replacement therapy may affect lactate production and clearance [1].

Clinical relevance and utility of lactate monitoring

Indications for Lactate Monitoring in Intensive Care

Lactate monitoring is crucial in managing critically ill patients, offering vital prognostic information and guiding resuscitation and treatment. Elevated blood lactate levels (hyperlactatemia) are common in critically ill patients and are a strong predictor of morbidity and mortality. The presence of increased lactate levels has significant implications for the morbidity and mortality of hyperlactatemic patients [10]. Lactate is typically measured to detect tissue hypoperfusion or inadequate tissue oxygenation. However, other processes unrelated to tissue oxygenation may also elevate lactate levels, especially in critically ill patients, where increased glycolysis may be a significant cause. Two recent studies have highlighted the importance of monitoring lactate levels and adjusting treatment based on changes in lactate levels during early resuscitation. Structured lactate measurements should be incorporated into resuscitation protocols, as lactate levels can be rapidly measured at the bedside from various sources [11]. Lactate monitoring can provide information about the development of organ failure, the duration of mechanical ventilation, the use of inotropes/vasopressors, the need for renal replacement therapy, and the length of ICU stay. Lactate monitoring is essential in managing critically ill patients, providing critical prognostic information, and guiding resuscitation and treatment. A careful interpretation of lactate levels and other physiological parameters is crucial for optimizing patient outcomes [12].

Relationship Between Lactate Levels and Patient Outcomes

Elevated blood lactate levels (hyperlactatemia) are common in critically ill patients and are a strong predictor of morbidity and mortality. Lactate is an important biomarker indicating inadequate tissue oxygenation, increased glycolysis, or impaired lactate clearance. Two recent studies have demonstrated that incorporating lactate-directed resuscitation therapy can improve clinical outcomes for critically ill patients. Lactate levels need to be checked by monitoring venous oxygen saturation to better guide diagnosis and treatment decisions [13]. Increased lactate levels can result from both anaerobic and aerobic mechanisms, so it is crucial to consider the underlying cause rather than just the lactate value. A significant relationship between lactate and pH only exists at higher lactate levels, making "lactate-associated acidosis" more appropriate than "lactic acidosis." Lactate levels can act as a "warning signal" to clinicians that a patient is not improving or may be deteriorating, even if other hemodynamic parameters appear stable [14]. Machine learning models have shown a promising ability to predict subsequent changes in serum lactate levels in critically ill patients. This could help alert clinicians to intensify care and guide the frequency of lactate monitoring. Higher lactate levels are associated with increased short-term (three to 30 days) and long-term (one year) mortality in critically ill patients, even at lactate levels within the normal range. In elderly patients with sepsis, a non-linear positive relationship was found between serum lactate and 28-day mortality, with a turning point at 5.7 mmol/L. Lactate levels ≥8.0 mmol/L were associated with significantly higher 30-day mortality in non-trauma critically ill patients in the emergency department [15].

Methods of lactate measurement

Different Techniques and Technologies Used for Lactate Measurement

The gold standard for lactate measurement is enzymatic assays, which use the enzyme lactate oxidase or LDH to quantify lactate concentrations. These assays can be performed on blood plasma and whole blood samples, providing accurate and reliable lactate measurements. However, enzymatic assays require specialized laboratory equipment and expertise [16]. Another accurate method for measuring lactate levels, particularly in research settings, is high-performance liquid chromatography (HPLC). HPLC can separate and quantify lactate from other compounds in biological samples. While HPLC-based lactate measurement is more complex and time-consuming than enzymatic assays, it offers excellent precision [17]. In contrast to these laboratory-based techniques, portable, handheld lactate meters are increasingly used for rapid, bedside lactate testing. These point-of-care devices measure lactate electrochemically or enzymatically in small whole-blood samples, providing quick results. However, point-of-care lactate meters may be less accurate than laboratory-based enzymatic assays, especially at higher lactate levels [18]. Emerging technologies are also expanding the options for continuous and non-invasive lactate monitoring. Microneedle biosensor patches, for example, can provide minimally invasive, real-time monitoring of lactate levels in interstitial fluid. These biosensors offer advantages over intermittent blood sampling, especially in settings with limited access to laboratory services. Optical and electrochemical sensors are also being developed for non-invasive, continuous lactate monitoring using techniques like Raman spectroscopy and enzymatic electrodes. However, they may have lower accuracy than invasive methods [19].

Pros and Cons of Each Method in Clinical Practice

Enzymatic assays are the gold standard for accurate and reliable lactate measurement in clinical practice. These assays use the enzyme lactate oxidase or LDH to precisely quantify lactate levels in both blood plasma and whole blood samples. The main advantage of enzymatic assays is their ability to provide highly accurate and reproducible results. However, the downside is that they require specialized laboratory equipment and expertise, and the results are not immediately available, as samples need to be processed [20]. In contrast, point-of-care lactate meters offer a more convenient option for rapid bedside testing. These handheld devices can measure lactate levels using only a small whole blood sample, providing quick results that can guide clinical decision-making in critical care settings. The main benefit of point-of-care meters is their ease of use and ability to deliver real-time lactate data. However, these devices may be slightly less accurate than laboratory-based enzymatic assays, particularly at higher lactate levels, and the accuracy can vary between different meter models [18]. HPLC is another method for measuring lactate, but it is primarily used in research settings rather than routine clinical practice. HPLC provides a highly precise quantification of lactate by separating and analyzing the compound from other substances in biological samples. While HPLC offers excellent accuracy, it is more complex and time-consuming than enzymatic assays, making it impractical for widespread clinical use [21]. Clinicians must weigh the trade-offs between accuracy, speed, and convenience when selecting a lactate measurement method. Enzymatic assays remain the gold standard for reliable lactate quantification, but point-of-care meters can be a valuable tool for rapid bedside testing. The choice of method should be guided by the specific clinical needs, available resources, and the need for precise versus timely lactate results [22].

Interpretation of lactate levels

Normal Versus Elevated Lactate Levels

An unstressed patient's normal blood lactate concentration is 0.5-1 mmol/L. Lactate levels may increase during physical activity or other stressful conditions when the body requires more energy [23]. Lactate levels in the range of 2-4 mmol/L are considered elevated or indicate hyperlactatemia. Lactate levels over 4 mmol/L are considered severe hyperlactatemia or lactic acidosis. Elevated lactate levels indicate an imbalance between lactate production and clearance and are associated with increased morbidity and mortality in critically ill patients [8]. Elevated lactate can be caused by tissue hypoxia, leading to anaerobic glycolysis and other processes unrelated to hypoxia, such as increased glycolysis. In critically ill patients, increased glycolysis may be a significant cause of hyperlactatemia rather than just tissue hypoxia. Other potential causes include liver dysfunction, sepsis, heart failure, respiratory failure, and metabolic disorders [11]. Elevated lactate has important prognostic implications, even if the underlying mechanism is unclear. Monitoring changes in lactate levels over time is crucial to guide resuscitation and treatment rather than relying solely on the absolute value. Lactate measurement should be accompanied by other markers like venous oxygen saturation to fully interpret the clinical significance [11].

Interpretation in Different Clinical Scenarios (e.g., Sepsis, Shock, Trauma)

Elevated lactate levels (>2 mmol/L) are a key diagnostic criterion for septic shock under the new definition. In sepsis, lactate levels correlate with disease severity, and mortality-risk levels of >4 mmol/L are associated with a 27% mortality rate. Lactate clearance (reduction in lactate over time) is an important prognostic factor, with <10% clearance in 6 hours predicting higher mortality. Lactate-directed resuscitation has been shown to improve outcomes compared to central venous oxygen saturation-guided therapy in sepsis [24]. Elevated lactate is also a marker of tissue hypoperfusion and anaerobic metabolism in other shock states, including cardiogenic, hypovolemic, and obstructive shock. Lactate levels correlate with shock severity levels; values >4 mmol/L indicate a severe shock with a high mortality risk. Monitoring lactate clearance can guide resuscitation and predict prognosis in shock similar to sepsis [13]. In traumatic injury, lactate is elevated due to tissue hypoperfusion and anaerobic metabolism. Lactate levels predict mortality risk in trauma patients in whom values >4 mmol/L are associated with a 33% mortality rate. Serial lactate measurements can monitor the adequacy of resuscitation and identify occult shock in trauma patients [25]. However, it's important to note that elevated lactate can occur in non-hypoperfusion states like liver disease, thiamine deficiency, and certain medications. Lactate should be interpreted in the clinical context, not in isolation. Factors like pH, anion gap, and clinical signs of shock should also be considered. Trending lactate levels over time are more informative than a single value, reflecting the balance between production and clearance [13].

Role of lactate monitoring in specific conditions

Sepsis and Septic Shock

Elevated lactate levels are a key indicator of tissue hypoperfusion and poor oxygen delivery in sepsis. Monitoring changes in lactate levels over time can help assess the severity of sepsis and guide resuscitation efforts. Lactate-directed resuscitation has improved outcomes in septic patients [26]. Persistently elevated or increasing lactate levels indicate poor perfusion and the need to escalate treatment, such as with fluid resuscitation and vasopressor therapy. Lactate measurement should be accompanied by venous saturation monitoring to fully assess tissue oxygenation and guide decision-making [27]. Machine learning models can help predict subsequent changes in serum lactate levels, aiding clinical decision support in sepsis management. By providing important prognostic information and guiding therapeutic interventions to improve tissue perfusion, lactate monitoring is a valuable tool in the early recognition of sepsis in patients and their resuscitation [28].

Cardiogenic Shock

Cardiogenic shock is a life-threatening condition characterized by inadequate cardiac output, leading to tissue hypoperfusion and organ dysfunction. It is typically triggered by severe cardiac dysfunction, most commonly due to acute myocardial infarction (AMI) [29]. The diagnosis of cardiogenic shock often occurs in emergency settings. Its symptoms include severe hypotension, cold and clammy skin, rapid heart rate, and altered mental status. Diagnostic tests to determine cardiogenic shock include blood pressure measurement, electrocardiogram (ECG), chest X-ray, blood tests, echocardiogram, and cardiac catheterization [30]. The immediate treatment of cardiogenic shock involves emergency life support, such as oxygen therapy, mechanical ventilation, and intravenous fluids to maintain blood pressure and cardiac output. Medications are also used, including vasopressors to increase blood pressure and inotropic agents to improve heart function [31]. The management of cardiogenic shock requires a multidisciplinary approach. This includes resuscitation and ventilation, hemodynamic support with devices like intra-aortic balloon pumps and extracorporeal membrane oxygenation, revascularization through percutaneous coronary intervention or coronary artery bypass grafting, and mechanical circulatory support devices [32]. Prevention of cardiogenic shock involves early recognition and treatment of the underlying conditions, such as the careful use of beta-blockers and angiotensin-converting enzyme (ACE) inhibitors in at-risk patients. Despite advances in treatment, the mortality rate for cardiogenic shock remains high, with survival rates around 50% at 30 days following diagnosis. However, timely diagnosis and multidisciplinary management have shown favorable effects on outcomes, highlighting the importance of early and appropriate intervention [32].

Trauma and Major Surgeries

Elevated blood lactate levels (hyperlactatemia) are common in critically ill trauma and surgical patients and indicate increased morbidity and mortality. Lactate is a critical prognostic biomarker, offering insights into disease severity and treatment efficacy. Monitoring changes in lactate levels over time is crucial for guiding resuscitation and treatment [33]. In trauma patients, persistently elevated or increasing lactate levels suggest inadequate perfusion and necessitate escalated treatment. Failure to normalize lactate is a strong negative prognostic indicator in post-injury patients and sustained elevated lactate levels have nearly seven times higher mortality compared to those whose lactate levels normalize. Incorporating blood lactate levels into predictive algorithms at the scene significantly enhances the ability to predict the need for immediate intervention in trauma patients experiencing hemorrhage. Lactate-directed resuscitation strategies have demonstrated improved outcomes in trauma settings [34]. In major surgery, elevated lactate levels indicate compromised tissue perfusion and impaired oxygen delivery. Serial lactate measurements are valuable in guiding fluid resuscitation and vasopressor therapy during the perioperative phase. Persistent elevation or an upward trend in lactate levels necessitates further optimization of hemodynamics and oxygen delivery strategies [13]. The role of lactate in different clinical situations is shown in Table 1.

**Table 1:** The role of lactate in different clinical situations

Situation/Condition	Role of Lactate	Clinical Implications
Sepsis	Elevated lactate levels indicate tissue hypoperfusion and hypoxia	Used as a marker for disease severity and prognosis
Shock (e.g., septic, hypovolemic)	Elevated due to increased production and decreased clearance	Used to monitor response to treatment and guide fluid resuscitation
Critical illness	Lactate levels can reflect the balance between lactate production and clearance	High levels are associated with poor outcomes and need intensive monitoring
Exercise	Produced during anaerobic metabolism; reflects muscle performance and fatigue	Useful for assessing athletic performance and recovery
Diabetes mellitus	Elevated lactate levels may indicate metabolic decompensation	Monitoring lactate can help in managing severe metabolic states
Liver disease	Impaired lactate clearance due to liver dysfunction	Can be used to evaluate liver function and disease severity
Respiratory diseases	Elevated lactate due to hypoxia and compromised oxygen delivery	Helps in assessing the severity of respiratory conditions

Lactate-guided therapeutic interventions

Strategies for Using Lactate Levels to Guide Treatment Decisions

One important strategy is to employ lactate-guided therapeutic interventions. A randomized trial demonstrated that aiming to reduce lactate levels by 20% every 2 hours during the first 8 hours of ICU admission resulted in decreased in-hospital mortality and shorter ICU stays compared to standard care. Interestingly, although the lactate-guided therapy group received more fluids and vasopressors, their reduction in lactate levels did not occur more rapidly than in the control group. This suggests that monitoring trends in lactate levels can indicate a response to resuscitation efforts, even if lactate levels do not decrease as quickly as targeted [35]. When interpreting lactate levels, it's crucial to recognize that increased lactate can stem from both anaerobic production and aerobic mechanisms, as well as changes in lactate clearance. Categorizing lactate levels into normal (<2 mmol/L), mild (2-4 mmol/L), and severe (>4 mmol/L) groups provides essential prognostic information to guide treatment decisions [13]. In addition to intermittent lactate measurements, continuous and non-invasive lactate monitoring shows promise and may reveal important dynamic changes. Integrating real-time lactate and glucose monitoring could also offer valuable insights into critically ill patients' liver function and metabolic status [36]. The evidence supports that early, targeted interventions to reduce elevated lactate levels can enhance clinical outcomes in intensive care settings, even if the lactate clearance rate does not differ significantly from standard care. Lactate monitoring thus provides critical prognostic information and guides resuscitation efforts in this high-acuity environment [37].

Evidence Supporting Lactate-Guided Therapy

Improved Clinical Outcomes: In a randomized trial, implementing therapy to reduce lactate levels by 20% every 2 hours during the first 8 hours of ICU admission decreased in-hospital mortality and shorter ICU stays than standard care. When adjusted for predefined risk factors, hospital mortality was significantly lower in the lactate-guided therapy group (HR 0.61; 95% CI 0.43-0.87) [35].

Faster Clinical Improvement: Patients receiving lactate-guided therapy exhibited lower SOFA scores between 9 and 72 hours, achieved earlier cessation of inotropes and were weaned from mechanical ventilation and discharged from the ICU sooner [35].

Limitations: Despite the lactate-guided therapy group receiving more aggressive fluid resuscitation and earlier initiation of vasopressors, no difference in the rate of lactate reduction was observed in the control group. This suggests that trending lactate levels may be an important indicator of response to resuscitation efforts, even if absolute lactate levels do not decrease as rapidly as targeted [35].

Conclusion: The findings of this randomized trial provide robust evidence that integrating lactate monitoring into resuscitation protocols, to reduce levels by 20% every 2 hours, can significantly enhance clinical outcomes in critically ill patients with elevated lactate upon ICU admission. While the exact mechanisms remain unclear, these outcomes were consistently observed across multiple endpoints [35].

Challenges and limitations

Common Pitfalls in Lactate Interpretation

Interpreting lactate levels involves avoiding key pitfalls that can lead to misinterpretation. Firstly, elevated lactate levels can stem from both anaerobic and aerobic mechanisms and changes in lactate clearance and may not always signify tissue hypoxia or a critical condition [38]. Secondly, the term "lactic acidosis" is often misapplied, as the relationship between lactate and pH is significant only at very high lactate levels (>4 mmol/L); "lactate-associated acidosis" is a more accurate term [39]. Clinicians must also be cautious about overlooking lactate clearance, as changes in lactate levels reflect a balance between production and clearance, especially in shock states where ongoing hyperlactatemia or rising lactate may indicate reduced clearance rather than increased production [13]. Furthermore, while elevated lactate is a robust predictor of morbidity and mortality, the degree of elevation does not always correlate with disease severity or prognosis; normolactatemia does not necessarily imply a non-severe condition [40]. Lastly, relying solely on lactate monitoring without considering other markers, such as venous oxygen saturation, can limit diagnostic accuracy and treatment decisions. Interpreting lactate trends requires a nuanced understanding and may benefit from input from clinicians experienced in managing critically ill patients [40].

Technical and Clinical Challenges in Lactate Monitoring

Lactate monitoring is crucial in intensive care, offering valuable prognostic insights and guiding resuscitation efforts. However, its use is fraught with challenges and limitations that impact interpretation and application in clinical settings. One of the primary challenges is the complexity of interpreting lactate levels. Elevated lactate can stem from both anaerobic and aerobic mechanisms and changes in lactate clearance, complicating the determination of its underlying cause. Moreover, the term "lactic acidosis" is often inaccurately used, as the significant relationship between lactate and pH is only evident at very high lactate levels, prompting the adoption of "lactate-associated acidosis" to better reflect clinical context [11].

Another significant hurdle is the development of continuous, non-invasive lactate monitoring technologies. While continuous monitoring promises real-time data, current systems face technical issues such as variations in blood flow affecting sensor accuracy, lag time in readings, and diffusion rate challenges. Microneedle-based systems offer a minimally invasive option but require careful management to ensure consistent sensor performance before and after insertion. Additionally, environmental factors like sweat and temperature can interfere with continuous lactate measurements, necessitating improved strategies to mitigate such interferences [41]. Integrating lactate monitoring into clinical decision-support systems remains another challenge. Although studies indicate the benefits of lactate-directed resuscitation therapy, the exact mechanisms underlying these benefits are still unclear. Lactate may serve more as an indicator of illness severity than a direct causal factor, complicating efforts to establish structured approaches for interpreting lactate changes in specific clinical scenarios. Addressing these challenges and limitations requires ongoing research and development efforts to refine lactate assessment methods and enhance their utility in managing critically ill patients [11].

Future directions and innovations

Emerging Trends in Lactate Monitoring Technologies

The field of lactate monitoring is evolving rapidly, and several exciting trends are emerging. One of the most promising developments is the advent of wearable devices capable of continuously monitoring lactate levels in sweat. These devices utilize advanced microfluidics, validated lactate biosensors, and integrated circuits for signal processing and wireless data transmission. These wearable technologies promise to reveal dynamic phenomena often missed with intermittent blood measurements by enabling real-time monitoring for athletes and critical care patients. Continuous, non-invasive lactate monitoring represents a significant advancement in the field [42]. Another noteworthy trend is the application of machine learning models to predict changes in serum lactate levels over time. Models like long short-term memory (LSTM) networks can accurately forecast lactate trends based on initial measurements and other clinical data. Integrating these predictive models into clinical decision support tools could facilitate more timely and appropriate interventions for patients at risk of lactate imbalance. As these predictive analytics become more sophisticated, they will be crucial in optimizing lactate-directed resuscitation strategies [15].

Multiparameter monitoring is also gaining traction, particularly in combining real-time lactate and glucose monitoring to provide comprehensive insights into liver function and metabolic status in critical care settings. Integrating lactate measurements with other physiological parameters such as pH, temperature, and oxygen saturation helps clinicians better interpret changes in lactate levels and make informed treatment decisions. Comprehensive multiparameter monitoring is expected to significantly enhance patient assessment and management [43]. Furthermore, there is a growing interest in incorporating lactate into mortality prediction scores such as APACHE and SAPS due to its strong prognostic value in critical illness. As evidence accumulates, structured decision support systems are likely to be developed to effectively interpret lactate changes in specific clinical contexts. This approach aims to prevent inappropriate clinical responses and ensure optimal utilization of lactate monitoring capabilities. The future of lactate monitoring appears promising, driven by innovative technologies poised to revolutionize clinical practice and improve outcomes for critically ill patients [44].

Potential Advancements in Lactate-Guided Therapy

The future of lactate-guided therapy in intensive care is poised for significant advancements, particularly in continuous, non-invasive lactate monitoring. Wearable devices designed to monitor lactate levels continuously in sweat are currently under development. These devices integrate advanced microfluidics, validated lactate biosensors, and circuits for signal processing and wireless data transmission. By enabling real-time monitoring, these wearable sensors hold promise for uncovering dynamic physiological phenomena often missed with intermittent blood measurements [45]. Predictive modeling and decision support systems are anticipated to play a pivotal role in the future of lactate-guided therapy. Machine learning models, such as those based on LSTM networks, have demonstrated the ability to accurately predict changes in serum lactate levels based on initial measurements and other clinical data. Integration of these predictive models into clinical decision support tools could facilitate timely and appropriate interventions for patients at risk of lactate imbalance, potentially enhancing the efficacy of resuscitation efforts and improving patient outcomes [46].

Advances in multiparameter monitoring are also expected to contribute significantly to the field. Combining real-time lactate and glucose monitoring can provide insights into critically ill patients' liver function and metabolic status. Additionally, integrating lactate measurements with other physiological parameters like pH, temperature, and oxygen saturation offers a comprehensive approach to better interpret changes in lactate levels and optimize treatment strategies. This holistic approach aims to improve diagnostic accuracy and treatment efficacy, thereby enhancing patient outcomes [43]. Furthermore, incorporating lactate data into prognostic scoring systems, such as APACHE and Simplified Acute Physiology Score (SAPS), will likely become standard practice due to lactate's strong prognostic value in critical illness. Developing structured decision support systems that effectively interpret lactate trends in specific clinical contexts can help mitigate inappropriate clinical responses and guide more informed therapeutic decisions. Ultimately, these advancements are expected to lead to more personalized and effective management of critically ill patients, improving overall patient outcomes [47].

## Conclusions

In conclusion, lactate monitoring is a cornerstone in managing critically ill patients in intensive care settings. Lactate levels provide critical information that guides clinical decision-making and therapeutic interventions by serving as a reliable biomarker of tissue perfusion and metabolic status. The comprehensive review of current literature underscores the significance of lactate as not merely an indicator of disease severity but also a predictor of outcomes in conditions such as sepsis, shock, and trauma. Continuing advancements in technology and research will likely refine our understanding of lactate dynamics and enhance its clinical utility. Embracing these insights is pivotal for optimizing patient care and improving survival rates and quality of life in critically ill populations.
